# *Post hoc* Bayesian model selection

**DOI:** 10.1016/j.neuroimage.2011.03.062

**Published:** 2011-06-15

**Authors:** Karl Friston, Will Penny

**Affiliations:** The Wellcome Trust Centre for Neuroimaging, University College London, Queen Square, London, UK

**Keywords:** Bayesian model evidence, Model selection, Automatic relevance determination, Savage–Dickey density ratio, Hyperparameters

## Abstract

This note describes a Bayesian model selection or optimization procedure for *post hoc* inferences about reduced versions of a full model. The scheme provides the evidence (marginal likelihood) for any reduced model as a function of the posterior density over the parameters of the full model. It rests upon specifying models through priors on their parameters, under the assumption that the likelihood remains the same for all models considered. This provides a quick and efficient scheme for scoring arbitrarily large numbers of models, after inverting a single (full) model. In turn, this enables the selection among discrete models that are distinguished by the presence or absence of free parameters, where free parameters are effectively removed from the model using very precise shrinkage priors. An alternative application of this *post hoc* model selection considers continuous model spaces, defined in terms of hyperparameters (sufficient statistics) of the prior density over model parameters. In this instance, the prior (model) can be optimized with respect to its evidence. The expressions for model evidence become remarkably simple under the Laplace (Gaussian) approximation to the posterior density. Special cases of this scheme include Savage–Dickey density ratio tests for reduced models and automatic relevance determination in model optimization. We illustrate the approach using general linear models and a more complicated nonlinear state-space model.

## Introduction

This paper is about optimizing or selecting among a large number of models using their Bayesian model evidence. It addresses the class of problems, under which different models can be formed by changing prior beliefs about their parameters; for example, by switching off various parameters or by changing their prior variance. The main point made in this paper is that it is only necessary to fit or invert a full model to access the model evidence or marginal likelihood (and the posterior density on the parameters) of any reduced model. This can greatly finesse the scoring of models, when exploring large model spaces in a *post hoc* fashion.

Conceptually, this treatment of model evidence highlights the connection between Bayesian model selection and the optimization of a (full) model, in terms of its priors. In both cases, one is maximizing model evidence by changing hyperparameters that encode the prior density over the parameters of a likelihood function. Operationally this is a form of empirical Bayes, in the sense that the priors are optimized using observed data. As such, it rests upon an implicit or explicit hierarchical structure in the form of the prior. This perspective unifies a number of model optimization and selection schemes, including parametric empirical Bayes (Efron and Morris, 1973; Kass and Steffey, 1989), automatic relevance determination (Mackay and Takeuchi, 1996; Tipping, 2001) and Bayesian model comparison (Kass and Raftery, 1995; Penny et al., 2004). We will try to illustrate this perspective with a few toy examples. A real world application of this scheme can be found in the context of network discovery, using dynamic causal modeling in Friston et al (2010a).

In classical parametric statistics, it is standard practice to invert or optimize the parameters of a generative (observation) model and then interrogate reduced forms (e.g., using the extra sum of squares principle and *post hoc t*-tests) to test a series of null hypotheses entailed by model reduction. Generally, it is not necessary to re-fit the parameters for each inference about a reduced model (null hypothesis) in relation to a full model (alternate hypothesis). In what follows, we describe the same sort of procedure for Bayesian model inversion and ensuing inference on models. The underlying theory is simple and follows from well known results; here, we highlight its potential to finesse the computational burden associated with scoring large numbers of models. This search over models is becoming an increasingly important problem in data-mining and causal modeling in the biological sciences. The sorts of models we have in mind here are those in which priors are required to resolve ill-posed inverse problems (for example the electromagnetic inverse problem in source reconstruction of electrical signals from the brain) and generalized convolution or state-space models used to explain biological time-series. In many instances, one is interested in eliminating redundant or irrelevant model parameters to find an efficient and parsimonious explanation for how data are generated. In a Bayesian context, this usually appeals to maximizing model evidence, which subsumes both accuracy and complexity. This evidence maximization emerges in a number of guises; for example automatic model selection (Friston et al., 2007) and relevance determination (Mackay and Takeuchi, 1996; Tipping 2001), which aim to switch off or suppress irrelevant model parameters. Crucially, this suppression or elimination can be formulated in terms of priors on the free parameters of a model, where increasing the precision (inverse variance) of appropriate shrinkage priors effectively sets these parameters to zero. However, this is just a special instance of optimizing the priors of a model, in relation to model evidence. We exploit this by noting that the evidence *per se* can be derived relatively simply from the posterior density over model parameters, under uninformative priors.

This note comprises two sections: In the first, we present the assumptions and derivations that motivate the scheme. We start with some general assumptions about the existence of a full model, which shares the same likelihood with a set of reduced models with different prior densities. Under these assumptions, it is simple to derive the evidence of any reduced model as a function of the posterior and prior densities of the full model (to within an additive constant). In the limiting case, when a subset of parameters is fixed at zero, we recover the well known Savage–Dickey density ratio (Dickey, 1971; Verdinelli and Wasserman, 1995). Our particular focus will be on the expression for model evidence under Gaussian assumptions about the form of the posterior over model parameters. This Laplace assumption is particularly relevant for variational or ensemble learning schemes, which predominate in many practical modeling applications (Beal and Ghahramani, 2003; Friston et al., 2007). In this context, the model evidence and posterior density over its parameters reduces to a simple analytic function of the means and precisions of the full prior and posterior. In the second section, we illustrate the use of this expression when optimizing priors or selecting among models defined in terms of their priors. We will use three examples of increasing complexity. The first uses a simple general linear model and looks at optimizing the priors on precision parameters, to highlight the potential usefulness of this approach in model optimization. The second example turns to model selection and focuses on optimizing shrinkage priors on unknown parameters, to identify key combinations of explanatory variables and eliminate redundant parameters. Finally, we consider a more complicated simulation using a nonlinear state-space model to illustrate both model selection and optimization.

## Theoretical background

In this section, we outline the overall approach in terms of the assumptions that define the problem we are interested in. We try to relate the results to established procedures such as those based upon the Savage–Dickey density ratio. In the subsequent section, we apply these results to toy examples to illustrate their usefulness. We address the problem of scoring large numbers (thousands or millions) of models or exploring continuous model spaces. This problem is addressed by exploiting situations in which each model can be formed from a full model by changing the priors over its parameters. In brief, this means we can compute the evidence and posterior density over the parameters of any reduced model that is nested within a full model, given the evidence and posterior of a full model. This rests on the following arguments:

Let a generative model *m*_*i*_ ∈ M specify a joint density on the some data γ ∈ ℝ and their causes *ϑ* ∈ ℝ (model parameters), in terms of a likelihood and prior:

(1) $$p\left(y\text{,}\vartheta |m_{i}\right)=p\left(y|\vartheta ,m_{i}\right)p\left(\vartheta |m_{i}\right)$$

Where *p*(*ϑ*|*m*_*i*_) ≜ *p*_*m*_*i*__(*ϑ*) denotes a family of distributions over models. We assume the existence of a full model *m*_F_ ∈ M that satisfies the following conditions for all models considered

(2) $$m_{i}≺m_{\text{F}}\Leftrightarrow \{\begin{array}{l} p\left(y|\vartheta ,m_{i}\right)=p\left(y|\vartheta ,m_{\text{F}}\right)\\ \Omega_{i}\subset \Omega_{\text{F}}:p\left(\vartheta \in \Omega_{i}|m_{i}\right)>0 \end{array}$$

Here, Ω_*i*_ denotes the support of the prior of the *i*-th model and ∀*i*:*m*_*i*_ ≺ *m*_F_ are reduced versions of the full model. Note that all models share the same likelihood but differ in their priors. The second condition just ensures the existence of the density ratios used below. A simple example may clarify what reduced means in this context: Let *m*_*i*_ ∈ M denote the class of general linear models and let *ϑ* range over values of variances of noise terms and linear coefficients. We say that *m*_*i*_ ≺ *m*_*j*_ if, for every coefficient *ϑ*_*k*_, we have *p*(*ϑ*_*k*_|*m*_*i*_) = 0 when *p*(*ϑ*_*k*_|*m*_*j*_) = 0 (but not conversely) and that the probability of the data is the same under both models for any assignment of values to the parameters.

Eq. (2) is not saying anything very deep; it is just defining a set or space of reduced models that can be formed from a full model by collapsing the prior density over one or more parameters. This effectively converts free-parameters into known (reduced) parameters that usually have a prior mean of zero. Note that the number or dimensionality of the parameters is the same for all models: What distinguishes models is whether their priors allow specific parameters to take non trivial values. This definition of a reduced model means that model optimization (selection) can be cast as optimizing the priors over the parameters of the full model, where the optimum prior (model) maximizes the marginal likelihood or evidence:

(3) $$\begin{array}{c} p\left(y|m_{i}\right)=\int p\left(y|\vartheta ,m_{i}\right)p\left(\vartheta |m_{i}\right)\text{d}\vartheta \\ =p\left(y|m_{\text{F}}\right)\int p\left(\vartheta |y,m_{\text{F}}\right)\frac{p\left(\vartheta |m_{i}\right)}{p\left(\vartheta |m_{\text{F}}\right)}\text{d}\vartheta \end{array}$$

Here, we have used Bayes rule and the fact that the likelihoods of the reduced and full model are the same. Crucially, the marginal likelihood or evidence under the reduced model is just the evidence under the full model times the posterior expectation of the prior density ratio. This means the quantities required to evaluate the evidence of any reduced model are furnished by the inversion of the full model (namely its evidence *p*(*y*|*m*_F_) and posterior density *p*(*ϑ*|*y*, *m*_F_)).

The equivalence of the likelihood in Eq. (2) also allows us express the posterior under the reduced model in terms of the posterior under the full model

(4) $$p\left(\vartheta |y,m_{i}\right)=p\left(\vartheta |y,m_{\text{F}}\right)\frac{p\left(\vartheta |m_{i}\right)}{p\left(\vartheta |m_{\text{F}}\right)}\frac{p\left(y|m_{\text{F}}\right)}{p\left(y|m_{i}\right)}$$

In fact, Eq. (3) obtains from Eq. (4) by integrating both sides over the parameters. In general, this marginalization only needs to be over the subset of (reduced) parameters *ϑ*_*i*_ ⊂ *ϑ* for which the priors differ. Given a bipartition *ϑ* = {*ϑ*_*i*_, *ϑ*_*\i*_} where *p*(*ϑ*_*\i*_|*m*_*i*_) = *p*(*ϑ*_*\i*_|*m*_F_), we can write Eq. (3) as a Bayes factor (Kass and Raftery 1995):

(5) $$\begin{array}{c} \frac{p\left(y|m_{i}\right)}{p\left(y|m_{F}\right)}=\iint p\left(\vartheta_{\backslash i}|\vartheta_{i},y,m_{\text{F}}\right)p\left(\vartheta_{i}|y,m_{\text{F}}\right)\frac{p\left(\vartheta_{i}|m_{i}\right)}{p\left(\vartheta_{i}|m_{\text{F}}\right)}\text{d}\vartheta_{\backslash i}d\vartheta_{i}\\ =\int p\left(\vartheta_{i}|y,m_{\text{F}}\right)\frac{p\left(\vartheta_{i}|m_{i}\right)}{p\left(\vartheta_{i}|m_{\text{F}}\right)}\text{d}\vartheta_{i} \end{array}$$

This expression only involves integrating over the marginal densities of the reduced parameters. Note that Eq. (5) does not make any assumptions about the form of the prior densities, provided they satisfy Eq. (2). We can further simplify things when the reduced prior is a point mass, (delta function) *p*(ϑ_*i*_|*m*_*i*_) = *δ*(*θ*_*i*_) that fixes a subset of parameters to a particular value, *θ*_*i*_. In this case, Eq. (5) reduces to the well-known Savage–Dickey density ratio (usually considered when *θ*_*i*_ = 0)

(6) $$\frac{p\left(y|m_{i}\right)}{p\left(y|m_{\text{F}}\right)}=\frac{p\left(\vartheta_{i}=\theta_{i}|y,m_{\text{F}}\right)}{p\left(\vartheta_{i}=\theta_{i}|m_{\text{F}}\right)}$$

In other words, the Savage–Dickey density ratio is a special case of the reduced evidence ratio that obtains when the reduced prior shrinks to a point mass. Eq. (6) is sensible, in that a conditional density on the reduced parameters that is far from its prior expectation indicates the reduced parameters are needed to explain the data and the reduced model has relatively low evidence.

### Model optimization under the Laplace assumption

Verdinelli and Wasserman (1995) consider generalized Savage–Dickey density ratios using the above arguments from point of view of sampling approximations. Here, we consider Eq. (3) under the Laplace approximation to the posterior. This is a useful and generic approximation exploited in variational Bayes and related free-energy schemes (Beal, 1998; Beal and Ghahramani 2003; Friston et al., 2007). In these schemes, a variational density *q*(ϑ|*m*_F_) is optimized with respect to a free-energy bound on the log-evidence:

(7) $$\begin{array}{c} F(y,q)=\ln p\left(y|m\right)-D(q\left(\vartheta |m\right)||p\left(\vartheta |y,m\right)\\ =\int q\left(\vartheta |m\right)\ln p\left(y|\vartheta ,m\right)d\vartheta -D\left(q\left(\vartheta |m\right)||p\left(\vartheta |m\right)\right) \end{array}$$

Here, D denotes Kullback–Leibler divergence. Maximizing free-energy makes it an approximation to the log-evidence and makes the variational density an approximate posterior. The second equality expresses free-energy as a mixture of accuracy (expected log-likelihood) and complexity (divergence between the posterior and prior). This means the model with the greatest free-energy is the most parsimonious but accurate explanation for the data; see Penny et al. (2004). There are numerous schemes that use this approach. We use it extensively under the Laplace assumption, with log-normal forms for non-negative scale parameters (e.g., Friston et al., 2003; Friston et al., 2007).

Our focus here is not on these variational schemes but on how to exploit their outputs; namely, the (approximate) log-evidence F(*y*, *q*) ≈ ln *p*(*y*|*m*) and (approximate) posterior *q*(*ϑ*|m) ≈ *p*(*ϑ*|*y*, *m*) of a model. From now on, we will assume the posterior and prior are Gaussian densities (this is known as Variational Laplace)

(8) $$\begin{array}{l} q\left(\vartheta |m_{i}\right)=N\left({\text{μ}}_{i},C_{i}\right):C_{i}=P_{i}{}^{-1}\\ p\left(\vartheta |m_{i}\right)=N\left({\text{η}}_{i},\Sigma_{i}\right):\Sigma_{i}=\Pi_{i}{}^{-1} \end{array}$$

In this context, we have remarkably simple expressions for the log-evidence and posterior for any reduced model in terms of the sufficient statistics of a full model

(9) $$\begin{array}{c} \frac{p\left(y|m_{i}\right)}{p\left(y|m_{\text{F}}\right)}=\int p\left(\vartheta |m_{i}\right)\frac{q\left(\vartheta |m_{\text{F}}\right)}{p\left(\vartheta |m_{\text{F}}\right)}\text{d}\vartheta \\ \Rightarrow \\ F_{i}=\frac{1}{2}\ln\frac{\left|\Pi_{i}\right|\left|P_{\text{F}}\right|}{\left|P_{i}\right|\left|\Pi_{\text{F}}\right|}-\frac{1}{2}\left(\mu_{\text{F}}^{\text{T}}P_{\text{F}}\mu_{\text{F}}+\eta_{i}^{\text{T}}\Pi_{i}\eta_{i}-\eta_{\text{F}}^{\text{T}}\Pi_{\text{F}}\eta_{\text{F}}-\mu_{i}^{\text{T}}P_{i}\mu_{i}\right)+F_{\text{F}}\\ \begin{array}{l} q\left(\vartheta |m_{i}\right)=N\left(\mu_{i},C_{i}\right)\\ P_{i}=P_{\text{F}}+\Pi_{i}-\Pi_{\text{F}}\\ \mu_{i}=C_{i}\left(P_{\text{F}}\mu_{\text{F}}+\Pi_{i}\eta_{i}-\Pi_{\text{F}}\eta_{\text{F}}\right) \end{array} \end{array}$$

Eq. (9) says that the posterior precision of the reduced model is the posterior precision of the full model minus the difference between the full and reduced precisions. The posterior expectation is a mixture of precision-weighted expectations. Note that when a parameter is removed from the model, by shrinking its prior variance to zero, the prior and posterior moments become the same and the parameter no longer contributes to the reduced free-energy.

Crucially, we can now compare two models in terms of their log-evidence, F_*i*_ − F_*j*_. This is a log-Bayes factor and is usually considered significant if it exceeds three (i.e., an odds ratio of about twenty to one). Alternatively, we can optimize the prior (and associated posterior) explicitly, with respect to the log-evidence in Eq. (9). Furthermore, we can consider any hyperparameterization of the prior *p*(ϑ|*m*(*λ*)) = *N*(*η*(λ), ∑(*λ*))) that induces a model. Here the hyperparameters *λ* control the prior mean *η*_*i*_ ≜ *η*(*λ*) and precision *Π*_*i*_ ≜ *Π*(*λ*) to produce a log-evidence, $F_{i}≜F(\lambda )$. This perspective takes us away from the notion of discrete models *m*_*i*_ : *i* ∈ ℕ and into a model space supported by the hyperparameters, *m*(*λ*): *λ ∈* ℝ. In this context, the optimum model and posterior are:

(10) $$\begin{array}{c} \lambda^{*}=\underset{\lambda }{\arg\max}F(\lambda )\\ q\left(\vartheta |m^{*}\right)=N(\mu^{*}\text{,}\;C^{*})\\ P^{*}=P{\;}_{\text{F}}+\Pi (\lambda^{*})-\Pi_{\text{F}}\\ \mu^{*}=C^{*}(P_{\text{F}}\mu_{\text{F}}+\Pi (\lambda^{*})\eta (\lambda^{*})-\Pi_{\text{F}}\eta_{\text{F}}) \end{array}$$

In what follows, we will illustrate both perspectives; namely, model selection, *i*^∗^ = arg max_*i*_ F_*i*_ and optimization, *λ*^∗^ = arg max_*λ*_ F(*λ*) and try to connect these to automatic relevance determination (ARD) and related schemes.

## Some examples

In this section, we use some simulated examples to illustrate the use of the scoring method described in the previous section. This section illustrates model optimization, in terms of optimizing the sufficient statistics or hyperparameters of prior densities, and model selection, by searching over large model spaces.

### Model optimization

We start with a very simple example; namely the optimization of the priors on the precision parameters of a general linear model. To illustrate this, we formed simulated data (response variables) by adding four regressors sampled from the normal distribution.

(11) $$\begin{array}{c} y=X\beta +\varepsilon \\ X\in R^{64\times 4}:X_{\mathrm{ij}}\sim N\left(0,1\right)\\ \beta \in R^{4\times 1}:\beta_{j}=1:j=1,\ldots ,4\\ \varepsilon \in R^{64\times 1}:\varepsilon_{i}\sim N(0,\exp(-\gamma_{j})):j=1,2 \end{array}$$

After adding random noise with a log-precision of two to the first half of the data and a log-precision of one to the second half, we then estimated the noise precision, allowing for different precisions over the first and second half of the observations. This model has six parameters, four regression parameters *β* ⊂ *ϑ* and two log-precision *γ* ⊂ *ϑ* parameters. These parameters were estimated using a standard variational EM scheme (Dempster et al., 1977), as described in Friston et al. (2007). Crucially, the posterior density on all parameters was assumed to be Gaussian. As is usual in these Variational Laplace schemes, we assumed the posterior density over the log-precisions is Gaussian (i.e., posterior precisions have a log-normal form). In short, we estimated the regression coefficients and log-precisions to provide a Gaussian posterior. The priors on the parameters were uninformative Gaussian shrinkage priors with a mean of zero and variance of 32.

**Fig. 1:**
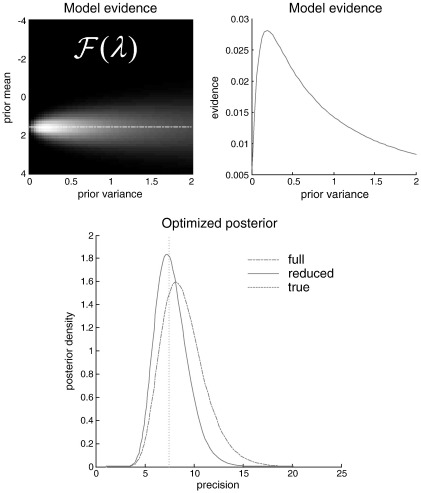
Model evidence and posterior densities on the precision parameters of a general linear model. Upper left: the (exponential of the) free-energy bound on log-evidence as a function of prior mean and variance of the log-precision parameters of a general linear model. Lighter areas denote higher evidence. The dashed line represents the optimum prior mean that maximizes evidence. Upper right panel: this shows the model evidence as a function of prior variance at the optimum prior mean. Lower panel: this shows the posterior density on the first of two precision parameters. The solid line shows the (optimized) posterior, based upon the optimum priors, using Eq. (10) in the main text. The broken line represents the same quantity but under the full priors. The vertical doted line corresponds to the value (precision) of observation noise used to generate the data.

After model inversion (using the Variational Laplace scheme described in Friston et al., 2007), we evaluated the model evidence under different priors on the log-precisions *P*(*γ*_*j*_|*m*(*λ*))=N(*λ*_1_, *λ*_2_). Fig. 1 (upper left panel) shows the log-evidence profile over the range of prior expectations and variances (*λ*_1_, *λ*_2_) we considered. It can be seen that the model evidence is greatest with a prior mean of just below two and a variance of about a quarter. This is shown more clearly in the upper right panel, which plots the model evidence (the normalized exponential of the free-energy approximation) as a function of prior variance for the optimum prior mean. If we now examine the implicit posterior from a model with these optimized priors (Eq. 10), one can see a characteristic shrinkage (increased precision) of the posterior to the optimized prior mean. The lower panel of Fig. 1 shows the posterior distribution over the first precision parameter for the full model and the optimized (reduced) model. In this example, the ensuing shrinkage has improved the posterior expectation, in relation to the true value (vertical line). Although this is not a very useful application of model optimization in a practical sense, it illustrates the notion of optimizing models through their priors and, implicitly, optimizing a posterior. There are two further points this example highlights:

First, the inversion scheme used to fit this model used a mean-field approximation that is ubiquitous in variational schemes. This assumes that the posterior over various subsets of parameters can be factorized. In this case, the factorization was between the regression and log-precision parameters; *q*(*β*, *γ*|*m*) = *q*(*β*|*m*)*q*(*γ*|*m*). Clearly this renders the posterior density an approximate density; however, it does not confound automatic model optimization. This is because the optimization of the priors on the log-precisions does not depend upon the posterior density over the regression parameters or posterior dependencies between the regression and log-precision parameters. This follows from Eq. (5), which shows that the model evidence depends only on the marginal densities of those subsets of parameters that are being optimized; in this instance, the log-precision parameters. This point holds true generally and may be of particular relevance for the large number of schemes that rest upon on a mean-field (conditional independence) assumption.

The second point is that this scheme allows one to optimize priors under any hyperparameterization. Indeed, it is the form of this hyperparameterization and the implicit constraints on the priors that make the optimization meaningful. This is meant in the sense that optimized priors are empirical priors, which benefit from formal constraints on the generative model. These constraints are implicit in the way the priors are hyperparameterized. This effectively adds a hierarchical level to the model, enabling further optimization of the model in relation to its evidence. In the example above, this hierarchical constraint was that, *a priori*, we believe that the two log-precisions are the same. In the absence of any constraints on the hyperparameterization of the priors, they would collapse to a point mass over their maximum likelihood value. This is intuitive, in the sense that empirical priors are informed by data and, in the absence of constraints, the best empirical estimate is the maximum likelihood. More formally, it is obvious from Eq. (7) that if there are no restrictions on the form of the optimum prior, it minimizes complexity when *p*(*ϑ*|*m*) = *q*(*ϑ*|*m*), leaving *q*(*ϑ*|*m*) free to maximize accuracy: accuracy is maximized when *q*(*ϑ*|*m*) has a point mass over the maximum likelihood. We will return to this in the discussion. In short, in the absence of constraints the best empirical prior is a point mass over the maximum likelihood value. This would have been the case had we hyperparameterized the prior on both log-precisions separately (results not shown). This selective collapse of prior densities is exactly the sort of behavior harnessed in model selection, which we turn to next.

### Model selection

Here, we focus on selecting among a number of discrete models using exactly the same approach as above but using a formally different hyperparameterization of the prior density. In the example below, prior variances can only take one of two values; zero or a fixed prior variance. One could regard this as a hyperparameterization of the prior covariance with switched variables along the leading diagonal.

(12) $$\begin{array}{c} p\left(\beta |m(\lambda )\right)=N(0,\Pi (\lambda ))\\ \Pi (\lambda )=daig(\gamma \lambda ):\lambda_{i}\in \{0,1\} \end{array}$$

Here, *γ* is the fixed prior variance. The objective here is to find the best permutation of zero and non-zero hyperparameters *λ* ∈ {0,1} that furnishes the greatest model evidence. We will illustrate this with another simple general linear model.

**Fig. 2:**
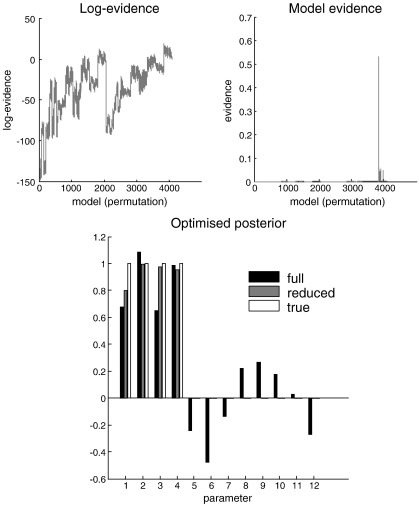
Model evidence and posteriors on the parameters of a linear model. Upper left panel: this shows the log-evidence over several thousand models that are distinguished by the permutation of priors on their regression parameters. These priors could take the value of zero or eight. Given there were twelve free regression parameters, this gives 2^12^ = 4096 models. Upper right: the same data as on the left but expressed in terms of evidence (the exponential of free-energy, normalized to a sum of one). Lower panel: the conditional means of the twelve parameters of this linear model. The black bars show the posterior means under the full model, the grey bars under a reduced model and the white bars show the true values. The key thing to note here is that (redundant) parameters have shrunk to zero, under the priors selected by automatic model selection.

In this example, we formed data by taking the sum of four regressors ***X*** ∈ ℝ^16 × 4^ drawn from the unit normal distribution as above and adding noise with a precision of two. We then inverted the model as above. Crucially, we added a further eight random regressors to the model before inversion. Effectively, this means we have to find a small number of relevant regressors (with non-zero regression parameters, *β* ∈ ℝ^12 × 1^) in a larger number. We do not known how many “needles” there are in this haystack but can use automatic model selection to find the optimum combination, in the hope of recovering the original four regressors. In this example, there are 4096 permutations of the hyperparameters, each corresponding to a different model. Although a large number, these models can be scored in under a second with an exhaustive *post hoc* search using Equation 9. The resulting log-evidences over all permutations of prior variances is shown in the upper left panel of Fig. 2, using *γ* = 8. The irregular profile of this scoring suggests that some regression parameters are more relevant than others. Crucially, there is a reasonably clear optimum model. This can be seen by reformulating the log-evidence in terms of model-evidence, shown on the upper right. Happily, the model selected was the true model in which the (first) four regression parameters had a non-zero prior variance, while the remaining (irrelevant) regressors were subject to very precise shrinkage priors. In terms of the posterior probability over models (under flat priors on models *per se*) we can be more than 50% confident that this is the most likely model. The lower panel of Fig. 2 shows the 12 regression parameter estimates in terms of their posterior mean for the full model, the reduced model and their true values. One can see immediately the benefit of model selection, in that the eight irrelevant parameters have been effectively switched off by very precise shrinkage priors. Interestingly, the four relevant parameters also improved, in terms of their distance from the true values. This reflects the fact that the optimized priors suppress irrelevant conditional dependencies among the posterior estimators.

Again, this is a rather trivial example that starts to get more interesting when considering ill-posed problems that call for some regularization or shrinkage priors. Although this example used 16 data-points and 12 regressors, exactly the same sorts of results obtain with underdetermined problems (results not shown). Because the scoring of each model is so quick, one can consider exhaustive searches of up to thousands or millions of discrete models.

**Fig. 3:**
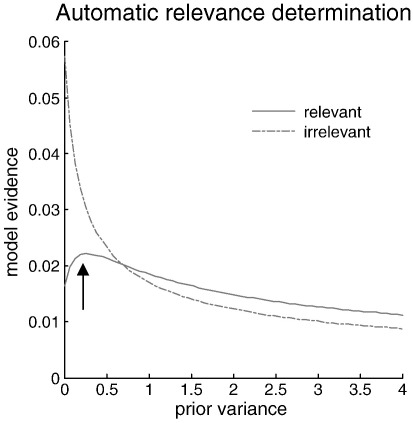
Model evidence as a function of prior variance for relevant and irrelevant parameters. This example comes from the inversion described in the previous figure and highlights the qualitative difference in the dependency of model evidence on prior variance. The important thing here is that only relevant parameters (that were used in generating data) have a maximum at a non-zero variance (marked with an arrow).

It is interesting to relate the automatic detection of relevant model parameters above to automatic relevance determination (ARD). Fig. 3 illustrates the basis of this ARD or switching off behavior in terms of the dependency of the evidence on the shrinkage priors of relevant and irrelevant parameters. The key thing to take from Fig. 3 is that the log-evidence for relevant parameters (here the first parameter) has a maxima at non-zero values. Conversely, the equivalent function for irrelevant parameters continues to increase as the prior variance approaches zero. This qualitative change in the points of inflexion induces a thresholding like behavior in the automatic model optimization, which explains the switching off of certain (irrelevant) parameters, when the maximum disappears. This behavior turns model optimization into categorical model selection. The irrelevant parameter here was the 8th regression parameter.

Automatic relevance determination (Mackay and Takeuchi, 1996; Tipping, 2001) is based on exactly the same model evidence maximization approach used above but calls upon particular forms for the prior densities that lead to sparse conditional means. Here, we were able to reproduce this automatic determination under the Laplace assumption, with a simple hyperparameterization of the priors on the model parameters. The reason that this works is because of the formal prior or constraint implied by the hyperparameterization; in which prior variances can only take one of two values. This gives the optimization of the hyperparameters the look and feel of a model selection procedure, as opposed to the optimization of continuous hyperparameters. The examples above highlight the deep connection between the optimization of the parameters of hierarchical generative models and the hyperparameters of non hierarchical models used here for model optimization and selection. In essence, all these procedures are trying to maximize the evidence for a model through placing formal constraints of a hierarchical sort on the model. In the final example, we will pursue both the model selection and optimization perspectives, in the context of a problem that has a growing and pragmatic appeal.

### Network discovery and automatic model selection

In this final example, we turn to a much more complicated generative model and a more specific sort of problem. The model that we use to generate data here is used as a generative model for brain imaging time series recorded from different parts of the brain. The problem that we are interested in is trying to discover the network of connections (i.e., an underlying dependency graph) that is responsible for observed brain responses. The details of the model we used (Friston et al., 2003) and the scheme we used to invert this model (Friston et al., 2010b) are not important here, because our focus is on how to use the posterior of a full model to discover the underlying network in terms of its adjacency matrix (the presence or absence of connections among observed brain regions). In brief, the model has a series of hidden neuronal and physiological states for each region *x*(*t*) ⊂ ϑ, whose dependencies are modeled using nonlinear random differential equations $\dot{x}(t)=f(x,\vartheta )+\omega$. These equations of motion mimic real physiological and neuronal processes in the brain and accommodate random or endogenous fluctuations $\tilde{\omega }\sim N\left(0,\Sigma \left(\vartheta \right)\right)$ on these states ($\tilde{\omega }={\left[\omega ,\omega^{\prime },\omega^{\prime \prime },\ldots \right]}^{T}$ denotes states in generalized coordinates of motion). The parameters of this model govern the dynamics intrinsic to each brain region and, crucially, the coupling between regions. These (extrinsic) between-region couplings are parameterized by a parameter matrix *A* ⊂ *ϑ* that can be viewed as a coupling matrix. This matrix plays the same role as a weighted adjacency matrix in graph theory, where a zero entry denotes no connection. The objective is to find the optimum adjacency matrix that specifies the underlying functional architecture; i.e., discover the network that explains the observed responses best. This example is used to show that automatic model selection works using a simplifying Laplace assumption, even with a highly nonlinear and dynamic (state-space) model with thousands of parameters (note the parameters include the generalized motions of all hidden states at each point in time).

**Fig. 4:**
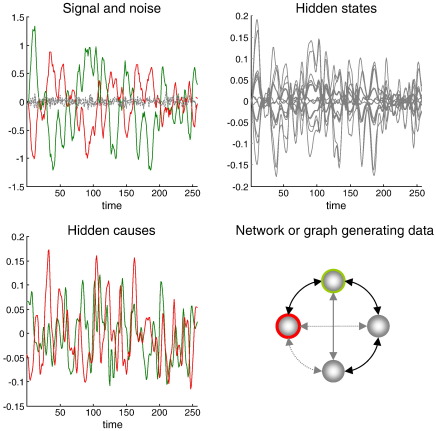
Example of synthetic data used for network discovery. Upper left panel: the simulated data over 256 (3.22 s) time bins comprising signal (solid lines) and correlated observation noise (broken lines). These simulated data were selected from two regions and were generated as a nonlinear function of region-specific hidden states shown on the upper left. These hidden states evolve dynamically according to equations of motion that model a physiological transduction of neuronal activity into measurable blood flow (hemodynamic) changes in the brain. The original perturbation to these dynamics arises from the hidden causes shown on the lower left. These were simply smooth random fluctuations sampled from a Gaussian distribution with a log-precision of eight. Examples of two hidden causes shown here correspond to the two colored regions in the graph (insert on the lower right). This graph depicts four nodes (brain regions) and all possible edges (putative connections). Hidden causes drive each of the four nodes to produce data. Crucially, the neuronal dynamics simulated in each node are communicated to other nodes through bidirectional connections (double headed arrows). When generating synthetic data we chose three out of a maximum of six connections. These are shown as solid arrows.

We generated synthetic brain responses by driving each of four nodes or regions with smooth random fluctuations (with a log-precision of six). The resulting neuronal fluctuations cause changes in hidden physiological states, both within each region and in other regions to produce observed (hemodynamic) signals of the sort measured in fMRI experiments. This signal is effectively a generalized convolution of the underlying neuronal activity, where the characteristic time constant of the implicit convolution kernel is about four seconds. Examples of these fluctuating inputs and the resulting signals are shown in Fig. 4. In addition, Fig. 4 (upper right panel) shows the hidden neuronal and physiological states that mediate between the hidden causes (fluctuating inputs) and signal (outputs). To generate these data, we used a simple (bidirectional) connectivity structure, where four regions (nodes) were coupled reciprocally in a chain (lower right insert). Data were generated over 256 time bins (each corresponding to 3.22 seconds of simulated time) and the model used to generate these data was inverted using Generalized Filtering and the usual Gaussian priors (see Friston et al., 2003). Generalized Filtering is a Bayesian filtering scheme in generalized coordinates of motion that retains the Laplace assumption but dispenses with mean field approximations (see Friston et al., 2010b). Further details about the generation and treatment of these sorts of synthetic data can be found in Friston et al. (2010a). Model inversion (Generalized Filtering) provided the posterior or conditional means and covariances for the coupling parameters, which entered Eq. (9) to furnish the free-energy and conditional moments of all reduced models.

**Fig. 5:**
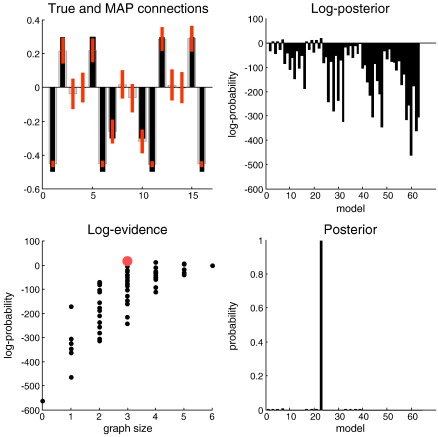
Results of model inversion and automated selection. Upper left panel: this shows the conditional means following inversion of the full model. The posterior means (grey bars) and 90% confidence intervals (red bars) are superimposed on the true values (black bars). It can be seen in most instances the true values fall within 90% confidence intervals. We have only shown the connections between brain regions in this figure; six of which were zero. Upper left: profile of log-evidences (or log-posterior of each model under flat model priors) over 64 models corresponding to different combinations of connections among the four nodes. Lower left: the same data but plotted as a function of graph size (number of bidirectional connections). The red dot corresponds to the model with the highest evidence, which was also the true model used to generate the data. Lower right: this portrays the same data as in the corresponding upper panel but here it is shown as a model posterior.

Fig. 5 shows the results of Generalized Filtering and subsequent model selection. The upper right panel shows the posterior density and true values of the (sixteen) connections among the four simulated brain regions. The right hand panels show the profile of log-evidences and evidences (i.e., the posterior probability of each model under flat model priors). It can be seen immediately that one model has been selected with nearly 100% posterior confidence. The model space here was created by considering all permutations of the prior variances on connections that could take values of zero or two. In the full model, all connections had a prior variance of two. There were only 64 such models because we included the additional constraint that if a connection existed in one direction, it should (*a priori*) exist in the other direction. This is a structural constraint that respects the known neuroanatomy of extrinsic connections in the brain. The real model had three bidirectional connections and, happily, was the model selected automatically. This example highlights the bilateral sensitivity of evidence to accuracy and complexity. The lower left panel of Fig. 5 shows the log-evidence of each of the 64 models grouped according to the number of connections (free coupling parameters). This is equivalent to graph size. It can be seen that, in general, as the number of parameters increases so does the evidence. This reflects the fact that the accuracy of the fit improves with the degrees of freedom that are endowed by additional coupling parameters. However, this increase in accuracy comes at a cost of complexity. Within each subset of models the most likely model (of graph size three or more used to generate the data) becomes unnecessarily complex when redundant connections are added and its evidence falls.

**Fig. 6:**
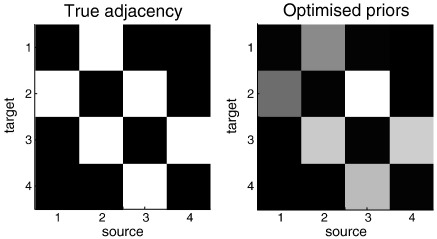
Adjacency matrices defining the connections between the four nodes in the simulated data of the previous figure. Left panel: this adjacency matrix defines a serially coupled chain with bidirectional connections and describes the connectivity used to generate data. Right panel: the optimized prior variance on each of the coupling parameters. This shows that the optimization of prior variance has identified the correct sparsity structure of connections and has assigned roughly equal prior variance to existing connections that were actually present. The gray scale is arbitrary.

Finally, to illustrate model optimization, we simply optimized the prior variance of each connection using Eq. (10) and a Gauss-Newton Scheme (as implemented in spm_argmax; http://www.fil.ion.ucl.ac.uk/spm). As anticipated, the prior variance collapsed to zero on connections that were absent, such that the optimized prior variance (organized as a weighted adjacency matrix) reflects the true connectivity structure (see Fig. 6). Note that in this hyperparameterization of the prior covariance, there was no formal constraint on reciprocal connections and yet a reciprocal connectivity has been selected automatically. In other words, we see automatic model selection emerging from optimization of hyperparameters that define a continuum of models (i.e., model space).

## Conclusion

In conclusion, we hope to have described an efficient *post hoc* scoring scheme based upon Bayesian model evidence. In essence, this scheme can be regarded as an add-on to any inversion scheme that can handle models with large numbers of unknown states and parameters. A particularly efficient version of *post hoc* model selection (optimization) obtains under the Laplace assumption. This is useful because the Laplace assumption is commonplace in many variational schemes of the sort illustrated above. Although not pursued here, *post hoc* model optimization provides an internal validation of the approximations implicit in variational schemes. This is because one can invert reduced models and ensure that the free-energy bound on log-evidence (and the approximate posterior densities) are roughly the same as those anticipated by inversion of a full model. We pursue this and empirical applications elsewhere, with a special focus on network discovery in the context of Dynamic Causal Modeling (Friston et al., 2010a). Another (practical) issue we have not pursued here is the pooling of evidence over units or subjects in group studies. The *post hoc* estimate of log-evidence (free-energy) in Eq. (9) can be treated in exactly the same way as in standard Bayesian model averaging or selection over subjects. For example, when treating models as fixed effects over subjects, the log-evidence inherent in multi-subject data is just the sum of log-evidences over subjects (under the assumption that subject-specific data are conditionally independent). This means that one can optimize models based on the summed log-evidence over subjects, following inversion of each subject's full model.

Although, in principle, *post hoc* model selection with the reduced free-energy finesses the computational problems of inverting large numbers of models, it still leaves open the problematic issue of searching over very large model spaces. For example, a dynamic causal model with eight nodes or sources has 2^8 × 8^ = 1.84 × 10^19^ permutations of connections that can be turned on or off. In our current implementation of *post hoc* model selection, we use a greedy search for very large model spaces. This entails identifying a subset of parameters, with the least evidence and searching over all reduced models within that subset. Redundant parameters and then removed and the procedure repeated until all model parameters have been considered or no further parameters can be removed. The appendix describes some of the pragmatic details, in terms of software notes for some of the key routines in SPM8.

The aim of this paper was to highlight the pragmatic utility of some simple results that follow from Bayes rule and show that the same sort of *post hoc* statistical model comparisons performed routinely in classical inference can be reproduced in a Bayesian setting. However, it struck us that the resulting scheme also provides a nice perspective on Bayesian model, optimization and selection: To reiterate our introductory comments; in both cases, one is maximizing the evidence by changing the hyperparameters of the prior density over the parameters of a likelihood function. Furthermore, as noted above, when there are no restrictions on the form of the empirical prior, it becomes the maximum likelihood (see Eq. (7)). In other words, this limiting case of model optimization is simply model inversion. This perspective unifies model inversion, optimization and selection schemes; including classical random effects modeling, parametric empirical Bayes (Efron and Morris, 1973; Kass and Steffey, 1989), automatic relevance determination (Mackay and Takeuchi, 1996; Tipping, 2001) and Bayesian model comparison (Kass and Raftery, 1995; Penny et al., 2004). The basic idea here is to recast any generative model in terms of parameters and hyperparameters and regard all model inversion, optimization and selection as maximizing the evidence with respect to the hyperparameters.

(13) $$\begin{array}{c} \lambda^{*}=\underset{\lambda }{\arg\max}p\left(y|m\left(\lambda \right)\right)\\ p\left(y,\vartheta |m\left(\lambda \right)\right)=p\left(y|\vartheta ,m\right)p\left(\vartheta |\lambda ,m\right) \end{array}$$

Here, the parameters can be thought of as the sufficient statistics of the likelihood function, while the hyperparameters become sufficient statistics of the prior on the parameters. Nearly all generative models and their optimization can be framed in this way. For example, take the hierarchical linear model underlying parametric empirical Bayes:

(14) $$\begin{array}{c} y=X^{\left(1\right)}\beta^{\left(1\right)}+\varepsilon^{\left(1\right)}\\ \beta^{\left(1\right)}=X^{\left(2\right)}\beta^{\left(2\right)}+\varepsilon^{\left(2\right)}\\ \vdots \\ \beta^{\left(n-1\right)}=X^{\left(2\right)}\beta^{\left(n\right)}+\varepsilon^{\left(n\right)}\\ \varepsilon^{\left(i\right)}\sim N(0,\Sigma (\gamma^{\left(i\right)}) \end{array}$$

Here, *X*^(i)^ *β*^(i)^ are linear mixtures of parameters that specify the form of the generative model. Eq. (14) can be written in terms of Eq. (13) as follows

(15) $$\begin{array}{c} p\left(y|\vartheta ,m\right)=N({\tilde{X}}^{\left(n\right)}\beta^{\left(n\right)},{\tilde{\Sigma }}^{\left(n\right)})\\ p\left(\vartheta |\lambda ,m\right)=p\left(\beta |\lambda ,m\right)p\left(\gamma |\lambda ,m\right)\\ p\left(\beta^{\left(n\right)}|\lambda ,m\right)=N\left(\mu_{\beta }^{\left(n\right)}\left(\lambda \right),\Sigma_{\beta }^{\left(n\right)}\left(\lambda \right)\right)\\ p\left(\gamma^{\left(i\right)}|\lambda ,m\right)=N\left(\mu_{\gamma }^{\left(i\right)}\left(\lambda \right),\Sigma_{\gamma }^{\left(i\right)}\left(\lambda \right)\right)\\ {\tilde{X}}^{\left(n\right)}=\prod_{i=1}^{n}X^{\left(i\right)}\\ {\tilde{\Sigma }}^{\left(n\right)}=\sum_{i=1}^{n}{\tilde{X}}^{\left(i-1\right)}\Sigma \left(\gamma^{\left(i\right)}\right){\tilde{X}}^{(i-1)T}:{\tilde{X}}^{\left(0\right)}=I \end{array}$$

This form shows that one has the latitude to optimize the model in terms of hyperparameters controlling the prior expectations μ_ϑ_^(*i*)^ (λ) or covariances Σ_ϑ_^(*i*)^ (λ) of the regression *β* ⊂ *ϑ* or precision (covariance) *γ* ⊂ *ϑ* parameters. For example, optimizing *μ*_*β*_^(*n*)^ (λ) with Σ_*β*_^(*n*)^ = 0 simply returns the maximum likelihood estimate of the parameters, while optimizing Σ_*β*_^(*n*)^ = (*λ*) with *μ*_*β*_^(*n*)^ = 0 optimizes their shrinkage priors (cf, ARD). Conversely, optimizing *μ*_*γ*_^(*i*)^ (*λ*) with Σ_*γ*_^(*i)*^ = 0, returns the restricted maximum likelihood (ReML) estimate of the covariance of random effects (Harville, 1977). Indeed, the ReML objective function is formally identical to the free-energy bound on log-evidence in Eq. (7) (Friston et al., 2007). Table 1 summarizes these and other examples. The point here is that, in principle, all these schemes could be implemented using Eqs. (9) and (10), under the Laplace assumption (following the inversion of a likelihood model with uninformative priors). We will pursue this in subsequent work.

As with all modelling initiatives, even exhaustive searches of model space will not disclose the optimum model, if the space does not include that model. For example, there could be unmodeled influences that, when included in the model, would increase its evidence. The scoring procedure described in this paper does not resolve the issue of how to define model spaces; it simply provides a computationally efficient way of searching those spaces, once they have been defined.

Evaluating the evidence for a model is the holy grail of most statistical and modeling endeavors. In this sense, the procedures described in this paper address an important problem. We have tried to stress their generality, with a particular emphasis on optimizing the priors of a model with respect to its reduced log-evidence or free-energy. This optimization goes much further than conventional uses of the underlying formalism, which are currently restricted to comparing models with and without various parameters, (e.g., with the Savage–Dickey density ratio). We have also emphasized the simplicity and efficiency with which one can score models under the Laplace assumption. However, this comes at a cost: for highly nonlinear models the true posterior density will not be Gaussian. This means that the free-energy will only bound log-evidence and may not be an accurate approximation. This raises two issues: First, is the free-energy a good approximation to the underlying log-evidence? Second, is the reduced free-energy a good proxy for the free-energy of reduced models? Clearly, these questions can only be resolved with access to the true posteriors and evidences. This is a focus of current work using Gibbs sampling. At present, our experience with mildly nonlinear models (such as those used in dynamic causal modeling of fMRI time series) suggests that the free-energy provides a reasonable approximation. However, this has yet to be established for more strongly nonlinear models. The second issue raises an interesting question. If the reduced free energy is not the same as the free energy of the reduced model, which is the best approximation? One might conjecture that the reduced free-energy may be a more reliable proxy for log-evidence because it is based on the free-energy of the full model, which may be less prone to reporting local minima. Clearly, to test this conjecture one needs the true log-evidence, which again speaks to the use of sampling approximations to the posterior densities. We are currently pursuing this. Our early impressions are that the reduced free-energy and free-energy of the reduced model are reasonably consistent for weakly nonlinear models and that the free-energy provides better approximations than other alternatives (such as the Akaike and Bayesian information criteria).

## Figures and Tables

**Table 1:** A brief summary of how various procedures can be cast as optimizing priors on model parameters, with respect to hyperparameters (sufficient statists of the prior density).

Hyperparameterization of prior	Procedure	Notes
*p*(*β* |*λ*, *m*) = N (*λ*, 0)	Maximum likelihood (ML)	*λ* ϵ ℝ^*N*^ become ML parameter estimates
$p\left(\gamma |\lambda \text{,}\;m\right)=N\left(\sum_{i}\lambda^{\left(i\right)}{\tilde{X}}^{\left(i-1\right)}{\tilde{X}}^{(i-1)T}\text{,}\;0\right)$	Restricted maximum likelihood (ReML)	*λ* ϵ ℝ^*M*^ become restricted ML covariance component estimates
*p*(*β* |*λ*, *m*) = N (*λ*, Σ_*β*_)	Maximum *a posteriori* (MAP)	*λ* ϵ ℝ^*N*^ become MAP estimators
*p*(*β* |*λ*, *m*) = N (0, *diag* (λ))	Automatic relevance determination (ARD)	*λ* ϵ ℝ^*N*^ optimize shrinkage priors on each parameter
*p*(*β* |*λ*, *m*) = N (0, *diag* (*λ* × Σ_*β*_))	Automatic model selection (AMS)	*λ* ϵ {0,1} switch off parameter combinations

These examples pertain to a generative model with *β* ϵ ℝ^*N*^ and *γ* ϵ ℝ^*M*^ parameters (sufficient statistics) specifying the mean and covariance a Gaussian likelihood, *p*(*γ*|*ϑ*, *m*) = N(*μ* (*β*), Σ(γ))*.* Here N(*λ*, 0) = *δ*(*λ*) denotes a point mass.

## Appendix and software notes

This appendix describes the key routines used in *post hoc* model selection and optimization currently available in our academic freeware. These are available as MATLAB scripts from (http://www.fil.ion.ucl.ac.uk/spm).

spm_dcm_post_hoc: *Post hoc* optimization (under Laplace approximation).

This routine searches over all possible reduced models of a full model (DCM) and uses *post hoc* model selection to select the best. Reduced models mean all permutations of free parameters (parameters with a non-zero prior covariance), where models are defined in terms of their prior covariance. The full model should be inverted prior to *post hoc* optimization. If there are more than 16 free-parameters, this routine will implement a greedy search: This entails searching over all permutations of the 8 parameters whose removal (shrinking the prior variance to zero) produces the smallest reduction (greatest increase) in model evidence. This procedure is repeated until all 8 parameters are retained in the best model or there are no more parameters to consider. When several DCMs are optimized together (as in group studies), they are checked to ensure the same free parameters have been specified and the log-evidences are pooled in a fixed effects fashion. The outputs of this routine are graphics reporting the model reduction (optimization) and an optimized DCM (for every input DCM) that contains reduced conditional parameters estimates.

This application of *post hoc* optimization assumes the DCMs that are optimized are the same model of different data. Normally, this would be a full model, in the sense of having the maximum number of free parameters, such that the set of reduced models is as large as possible. In contrast spm_dcm_search operates on different DCMs of the same data to identify the best model:

spm_dcm_search: *Post hoc* selection (under Laplace approximation)

spm_dcm_search operates on different DCMs of the same data to identify the best model. It will invert the full model, whose free-parameters are the union (superset) of all free parameters in each model specified. The routine then uses *post hoc* selection to evaluate the log-evidence (reduced free-energy) and conditional density over free-parameters of each model specified. The DCM specified does not need to be estimated: spm_dcm_search will invert the requisite (full DCM) automatically. The outputs of this routine are graphics reporting the model space search (optimization) and a structure containing the fields:

*DCM.P—character/cell array of DCM filenames*

*DCM.PF—their associated (reduced) free-energies*

*DCM.PP—and posterior (model) probabilities*

In addition, the reduced free-energies and posterior estimates of each model in *DCM.P* are saved for subsequent searches over different partitions of model space.

spm_dcm_optimise: Optimizes the priors of a model (under Laplace approximation)

This routine optimizes the prior covariance on the free parameters of any model (DCM) under the Laplace approximation. In other words, it assumes that the prior means are fixed and will maximize model evidence with respect to the hyperparameters of a function that returns the prior covariance. This optimization uses the reduced free-energy, based upon the posterior and prior densities of the full model supplied. If the prior covariance function is not specified, this routine will assume a simple diagonal form with a single hyperparameter. In principle, this routine can be used in a flexible and powerful way to emulate hierarchical modeling by using suitable prior covariance functions with unknown hyperparameters. The outputs are the prior moments (mean and covariance) of the optimum model.
